# Glutamate transporters: The arrestin connection

**DOI:** 10.18632/oncotarget.13999

**Published:** 2016-12-16

**Authors:** Francisco Zafra, Ignacio Ibáñez, Cecilio Giménez

**Affiliations:** ^1^ Centro de Biología Molecular Severo Ochoa, Facultad de Ciencias, Consejo Superior de Investigaciones Científicas, Universidad Autónoma de Madrid, Madrid, Spain

**Keywords:** intracellular trafficking, endocytosis, glutamate, ischemia, ubiquitination, transport, Neuroscience

The neurotransmitter glutamate is a major player in the physiology and pathology of the nervous system. Glutamate receptors are responsible for fast excitatory neurotransmission underlying the sensory, motor and cognitive functions of the nervous system. However, overstimulation of these receptors, and in particular those of the NMDA receptor subtype, is associated with neuronal death in acute lesions, like traumatic brain injury or stroke. Glutamate plays an important role in chronic diseases of the nervous system as well. For instance, glioma cells release glutamate which causes excitotoxic death to surrounding neurons, thereby vacating room for tumor expansion. Additionally, glutamatergic dysfunction contributes to the progression of neurodegenerative diseases like ALS, Huntington or Alzheimer diseases. Consequently, the precise control of the glutamate fluxes between neurons and glial cells is a pivotal issue in the physiopathology of the brain. Essentially, these fluxes are controlled by diverse glutamate transporters of the solute carrier 1 (SLC1) gene family, although SLC1A2 (named EAAT2 in humans and GLT-1 in rodents) accounts for most of the glutamate transport. This transporter is mainly expressed in astrocytes, but neuronal forms of the protein also exist. The concentration of GLT-1 on the cell surface is controlled by diverse regulatory mechanisms ranging from genetic and epigenetic control to trafficking events that adjust the concentration of the transporter on the cell surface to the specific requirements of the brain at a given moment. Recent evidence indicated that GLT-1 is mainly clustered in patches opposed to synaptic sites and that these clusters dissociate in response to neuronal activity, which promotes the lateral mobility of the transporter. The conformational changes induced by the presence of the substrate drives the dissociation of GLT-1 clusters on the surface of astrocytes [1]. Previous observations by our group indicated that a fraction of these GLT-1 molecules undergoes endocytosis to early/recycling endosomes, a process that relies on transporter ubiquitination initiated by the HECT ubiquitin ligase Nedd4-2 [2]. We have made further advances in understanding this process, discovering that the internalization of the transporters is triggered by the catalytic activity of the transporter itself induced by glutamate. This is achieved when glutamate reaches concentrations similar to those present in the extracellular milieu in ischemic or other traumatic events or even after intense glutamatergic activity [3]. Mechanistically, we have proved that the presence of glutamate promotes the recruitment of the endocytic adaptor β-arrestin 1 that in turn captures Nedd4-2 at the membrane, and that these two events precede and are necessary for the internalization of the transporter, since knocking down the expression of β-arrestin 1 suppressed both the ubiquitination and the intenalization of GLT-1.

Interestingly, the substrate-induced internalization mechanism mediated by HECT ubiquitin ligases and arrestins has been evolutionarily conserved at least since the emergence of eukaryotes. Many plasma membrane transporters in yeast are endocytosed in response to excess substrate or certain stresses using Rsp5 (orthologous of Nedd4-2) and arrestins [4]. It might seem paradoxical that the endogenous homeostatic environment of the brain conserves this regulatory process. However, as mentioned, brain homeostasis is sharply disrupted under diverse pathological situations, and perhaps the need to counteract this abrupt alteration of the environment has pushed for the conservation of the mechanism. During a brain ischemic episode, the temporal profile of the endocytic event suggests that decreasing GLT-1 concentration on the cell surface might tend to decrease the massive release of glutamate that, via the reverse mode of the transporter, occurs in the brain several minutes after the ischemic episode, when the ionic gradients that energize the transporter have dissipated as a consequence of ATP exhaustion [5]. Nevertheless, it cannot be excluded that the internalization of the transporter might be deleterious, contributing to the brain damage triggered by the ischemic accident. Futures studies are necessary to assess the physiopathological consequences of GLT-1 downregulation under excitotoxic conditions.

β-Arrestins are mainly known for their role in controlling G-protein coupled receptors (GPCRs). However, accumulating evidence places them as adaptors that control the trafficking of other non-GPCRs and different membrane proteins, acting as scaffold platforms where not only the ubiquination machinery converge, but other intracellular signaling pathways like c-Src, components of the MAP kinase cascade or GRK2. Since it is known that GLT-1 activity is controlled by ERK or Akt in astrocytes and neurons [6, 7], it is tempting to speculate that arrestins might play a role in connecting the subcelullar localization, stability and activity of the transporter with the activity of these signaling pathways. A detailed clarification of the mechanisms controlling the concentration of GLT-1 in the cell surface might provide novel potential therapeutic targets for pharmacological interventions in pathologies affecting fluxes of glutamate.
